# Tissue factor-dependent and -independent pathways of systemic coagulation activation in acute myeloid leukemia: a single-center cohort study

**DOI:** 10.1186/s40164-015-0018-x

**Published:** 2015-08-06

**Authors:** Christina Dicke, Ali Amirkhosravi, Brigitte Spath, Miguel Jiménez-Alcázar, Tobias Fuchs, Monica Davila, John L Francis, Carsten Bokemeyer, Florian Langer

**Affiliations:** II. Medizinische Klinik und Poliklinik, Hubertus Wald Tumorzentrum, Universitäres Cancer Center Hamburg (UCCH), Universitätsklinikum Eppendorf, Martinistr. 52, 20246 Hamburg, Germany; Florida Hospital Center for Thrombosis Research, 2566 Lee Road, Winter Park, FL 32789 USA; Institut für Klinische Chemie und Laboratoriumsmedizin, Universitätsklinikum Eppendorf, Martinistr. 52, 20246 Hamburg, Germany

**Keywords:** Acute myeloid leukemia, Tissue factor, Disseminated intravascular coagulation, Cell-derived microparticles, DNA

## Abstract

**Background:**

In acute myeloid leukemia (AML), disseminated intravascular coagulation (DIC) contributes to morbidity and mortality, but the underlying pathomechanisms remain incompletely understood.

**Methods:**

We conducted a prospective study on 69 patients with newly diagnosed AML to further define the correlates of systemic coagulation activation in this hematological malignancy. Tissue factor procoagulant activity (TF PCA) of isolated peripheral blood mononuclear cells (PBMCs) and TF expression by circulating microparticles (MPs) were assessed by single-stage clotting and thrombin generation assay, respectively. Soluble plasma TF antigen and secretion of vascular endothelial growth factor (VEGF) by cultured PBMCs were measured by ELISA. Cell-free plasma DNA was quantified by staining with a fluorescent dye.

**Result:**

TF PCA of PBMCs was significantly increased in AML patients as compared to healthy controls. Furthermore, TF PCA was significantly associated with decompensated DIC at presentation, as defined by a plasma fibrinogen level of ≤1 g/L (n = 11). In addition to TF PCA and circulating blasts, serum lactate dehydrogenase, a surrogate marker for leukemic cell turnover, correlated with plasma D-Dimer in the total patient cohort and was significantly increased in DIC patients, suggesting a role for myeloblast apoptosis/necrosis in activation of the TF-dependent coagulation pathway. Consistently, TF-bearing plasma MPs were more frequently detected and levels of soluble TF antigen were significantly higher in DIC vs. non-DIC patients. No association was found between TF PCA expression and VEGF secretion by isolated PBMCs, but significantly increased levels of cell-free plasma DNA pointed to a contribution of the intrinsic contact pathway to systemic coagulation activation in the total patient cohort and in patients with lower TF PCA expression. While PBMC-associated TF PCA had no effect on long-term survival, DIC occurrence at presentation increased the risk of early mortality.

**Conclusion:**

In newly diagnosed AML, TF expression by PBMCs and shedding of TF-bearing plasma MPs are central to the pathogenesis of DIC, but additional pathways, such as DNA liberation, may contribute to systemic coagulation activation.

## Background

Acute myeloid leukemia (AML) is a highly aggressive hematological malignancy characterized by clonal expansion of transformed myeloblasts. Patients with AML are at increased risk for both hemorrhagic and thromboembolic complications [1–3]. Besides disease- or treatment-related thrombocytopenia, AML patients may suffer from complex systemic coagulopathies such as overt disseminated intravascular coagulation (DIC), excessive fibrinolysis, or non-specific proteolysis. In particular, patients with acute promyelocytic leukemia (APL) are at substantial risk for a thrombohemorrhagic syndrome that, despite modern anti-leukemic therapy, still significantly contributes to early morbidity and mortality [4].

In newly diagnosed AML, potentially large numbers of myeloblasts circulate in direct contact with the plasma compartment. It is thus very likely that the procoagulant (or fibrinolytic) phenotype of AML blasts will have a profound effect on the hemostatic system. In fact, early studies have implicated over-expression of tissue factor (TF), the principal initiator of the extrinsic coagulation protease cascade, in the pathogenesis of AML-associated DIC [5–8]. More recent studies, however, have questioned the relevance of TF-driven coagulation activation to the hypercoagulable state in various hematological malignancies, including AML, and suggested alternative mechanisms such as acquired activated protein C resistance [9, 10].

Because a thorough understanding of the cellular and molecular pathways underlying AML-associated DIC is mandatory to identify novel drug targets for the prevention and treatment of potentially fatal thrombohemorrhagic events, we conducted a prospective study to further define the correlates of systemic coagulation activation in 69 patients with newly diagnosed AML.

## Results

### Patient population

The primary study population comprised 69 patients with newly diagnosed AML (Table 1). The mean age was 59 ± 16 years, and 61% of study subjects were males. While 56 patients (81%) had de-novo (i.e. primary) AML, including five patients with APL (M3), 13 patients (19%) had secondary AML resulting from either myelodysplastic syndrome (MDS) or myeloproliferative neoplasia (MPN).

**Table 1:** Demographic, clinical and laboratory characteristics of AML patients

	Normal range	Patients (n = 69)
Age (years)		59 ± 16
Gender		
Male		42 (61%)
Female		27 (39%)
AML		
Primary		56 (81%)
M0/M1/M2		32
M3 (APL)		5
M4/M5		19
M6/M7		0
Secondary		13 (19%)
Post MDS		12
Post MPN		1
Peripheral blasts (10^3^/µL)		12 (2–30)
Platelets (10^3^/µL)	150–400	47 (25–88)
Hemoglobin (g/dL)	12.3–15.3	9.6 ± 1.9
Prothrombin time (%)	80–130	79 ± 23
Fibrinogen (g/L)	1.8–4.0	4.3 ± 2.3
D-dimer (mg/L)	0.0–0.5	5.1 (1.6–22.1)
LDH (U/L)	84–246	474 (339–884)

Data are presented as mean ± standard deviation or as median and interquartile range. AML subtypes are according to the French–American–British (FAB) classification.

*APL* acute promyelocytic leukemia, *LDH* lactate dehydrogenase, *MDS* myelodysplastic syndrome, *MPN* myeloproliferative neoplasia.

### TF PCA of PBMCs is increased in AML

**Fig. 1:**
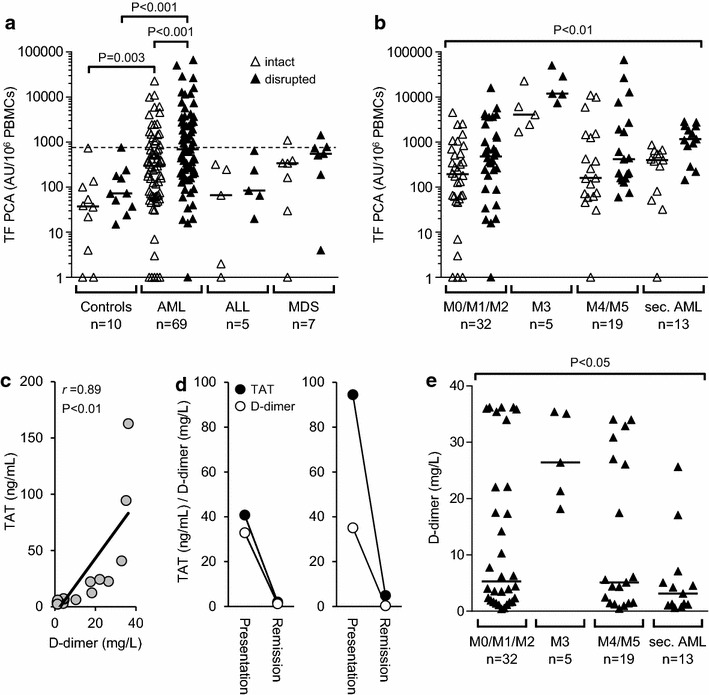
Expression of tissue factor-specific procoagulant activity (*TF PCA*) by peripheral blood mononuclear cells (*PBMCs*) and markers of systemic coagulation activation. **a** PBMCs were isolated from patients with newly diagnosed acute myeloid leukemia (*AML*), acute lymphoblastic leukemia (*ALL*), or myelodsyplastic syndrome (*MDS*) and analyzed for TF PCA, expressed as arbitrary units (*AU*), by single-stage clotting assay both before (*intact*) and after repeated freeze–thawing (*disrupted*). The *dashed line* indicates the upper limit of the reference range for TF PCA of intact PBMCs as determined by analysis of ten healthy controls. *P* values are according to Mann–Whitney U test. **b** Levels of PBMC-associated TF PCA according to FAB subtypes. *P* value is according to Kruskal–Wallis test. For illustration purposes only, data points with “zero” TF PCA were plotted as 1 AU/10^6^ PBMCs in *panels* **a** and **b**. **c** In a subgroup of 20 patients, plasma levels of both D-dimer and thrombin–antithrombin (*TAT*) complexes were measured at initial presentation. Correlation coefficient and *P* value are according to the method of Spearman. **d** In two patients, D-dimer and TAT plasma levels were measured at presentation and during AML remission. **e** D-dimer plasma levels according to FAB subtypes. *P* value is according to Kruskal–Wallis test.

We used a single-stage clotting assay to measure TF-specific procoagulant activity (TF PCA) in intact and disrupted PBMCs. In the total AML patient cohort (n = 69), TF PCA was significantly increased in disrupted as compared to intact PBMCs (median TF PCA, 696 vs. 293 AU/10^6^ cells, *P* < 0.001) (Fig. 1a), indicating that a significant proportion of the TF PCA was latent (or cryptic) on unperturbed cells. In five AML patients (7%), TF PCA was undetectable on intact PBMCs, but could be decrypted by cell disruption in four of them. In the remaining patient, however, TF PCA was absent both before and after cell disruption. In the 64 patients with measurable TF PCA on intact PBMCs, cell disruption resulted in an average 5.1-fold increase in cellular TF PCA. In the total study cohort (n = 69), there was a significant correlation between TF PCA of intact and disrupted PBMCs (*r* = 0.80; *P* < 0.001).

TF PCA of PBMCs was significantly increased in AML patients (n = 69) as compared to healthy controls (n = 10) (Fig. 1a), suggesting that leukemic transformation was associated with upregulation of the TF-dependent coagulation pathway. However, PBMC-associated TF PCA varied widely among study subjects with 50 AML patients (72%) having TF PCA levels within the reference range, the upper limit of which was defined as the 95th percentile of TF PCA levels of intact PBMCs within the control group. To compare PBMC-associated TF PCA expression in newly diagnosed AML with that in other hematological malignancies, we also studied patients with MDS or acute lymphoblastic leukemia (ALL). There were no statistically significant differences in median TF PCA levels between AML patients (n = 69) and patients with either MDS (n = 7) or ALL (n = 5), albeit none of the latter patients had a PBMC-associated TF PCA level of >1,500 AU/10^6^ cells (Fig. 1a). Collectively, these findings indicate that PBMC-associated TF PCA is overexpressed at least in a subgroup of patients with newly diagnosed AML.

### TF PCA expression among FAB subtypes

To analyze PBMC-associated TF PCA expression among subtypes of the French–American–British (FAB) classification, AML patients (n = 69) were grouped in those with M0/M1/M2 (n = 32), M3 (n = 5), M4/M5 (n = 19) or secondary AML (n = 13) (Fig. 1b). When compared to healthy controls, TF PCA levels of both intact and disrupted PBMCs were significantly increased in each of these subgroups [not shown]. When compared among each other, patients with APL (M3) had significantly higher TF PCA levels than patients included in the other subgroups, although values of PBMC-associated TF PCA were overlapping with those in the M0/M1/M2 and M4/M5, but not with those in the secondary AML subgroup (Fig. 1b).

### Markers of systemic coagulation activation

Because plasma D-dimer is readily available and widely used in clinical practice and part of the SSC/ISTH DIC score [11], this parameter was chosen to assess systemic coagulation activation in our study. However, D-dimer originates from plasmin-mediated degradation of cross-linked fibrin and therefore reflects fibrinolytic rather than procoagulant activity. In contrast, circulating thrombin–antithrombin (TAT) complexes are a more accurate marker of in vivo thrombin generation. To investigate the usefulness of D-dimer as a hemostatic activation marker in newly diagnosed AML, we additionally measured circulating TAT complexes in a subgroup of 20 AML patients, the selection of which was based on the degree of D-dimer elevation (low vs. high) and availability of plasma samples. In this subgroup, there was a quite strong correlation between the two parameters (Fig. 1c). Follow-up plasma samples were available from two patients achieving complete hematological remission after the first cycle of induction chemotherapy. Plasma levels of D-dimer and TAT complexes either normalized or decreased close to normal in both patients (Fig. 1d), further indicating that plasma D-dimer may be an appropriate marker of systemic coagulation activation in newly diagnosed AML, particularly in the absence of recent surgery, acute infections, and relevant organ dysfunctions, all of which were exclusion criteria in our study (see section “Methods”).

When analyzing the total AML patient cohort (n = 69), only two patients (3%) had D-dimer plasma levels within the reference range (i.e. <0.5 mg/L). Similar to PBMC-associated TF PCA, plasma D-dimer levels were highest in the M3 subgroup, but levels exceeding 30 mg/L were also observed in the M0/M1/M2 and M4/M5 subgroups (Fig. 1e).

### Role of cellular TF PCA in DIC evolution

Of the 69 AML patients, 11 (16%) presented with a (thrombo)hemorrhagic syndrome due to decompensated DIC, as defined by a plasma fibrinogen level of ≤1 g/L (referred to as DIC hereafter) (Table 2). In non-DIC patients (n = 58), the median plasma fibrinogen level was 4.7 g/L (range, 1.8–9.9 g/L). All DIC patients had primary AML, and APL (M3) was the diagnosis in four of them. Thus, one of the five APL patients included in this study had no DIC at presentation. Of the remaining seven DIC patients, three had acute (myelo)monocytic leukemia (M4/M5) and four had AML M1 or M2.

**Table 2:** Characteristics of patients with decompensated DIC at presentation

No.	AML subtype	Age (years)	Sex	PB (10^3^/µL)	Platelets (10^3^/µL)	Fg (g/L)	D-dimer (mg/L)	TF PCA (AU/10^6^ cells)	MP TF PCA	TF antigen (pg/mL)	DNA (ng/mL)	LDH (U/L)	Clinical outcome
1	M1	74	♀	287	25	0.8	36.2	1,483	No	n. d.	n. d.	924	Deceased after 3 days (fatal PE)
2	M3 (APL)	61	♀	97	14	0.8	26.4	6,141	Yes	n. d.	n. d.	n.d.	Deceased after 2 days (fatal ICH)
3	M5	69	♂	1	114	0.3	32.9	9,939	n. d.	32	220	1,663	Deceased after 4.3 months
4	M3 (APL)	56	♂	6	36	0.6	35.1	4,045	Yes	101	77	1,881	Alive at 69 months follow-up
5	M5	73	♂	1	43	0.6	34.1	11,070	No	40	38,716	6,841	Deceased after 6 days (fatal ACS)
6	M1	74	♀	40	88	0.6	22.1	4,515	Yes	115	1,513	1,409	Alive at 23 months follow-up
7	M2	69	♀	79	80	0.9	35.4	515	Yes	72	222	566	Alive at 52 months follow-up
8	M3 (APL)	47	♂	1	21	1.0	35.4	22,779	Yes	43	344	429	Deceased after 2.9 months (fatal pneumonia)
9	M3 (APL)	39	♀	89	25	0.8	21.3	1,693	n. d.	n. d.	n. d.	3,072	Deceased after 5 days (fatal ICH)
10	M2	70	♂	39	51	0.6	34.0	2,537	Yes	76	3,086	1,444	Deceased after 8.3 months
11	M4	21	♂	115	61	1.0	34.0	5,926	No	141	450	2,140	Deceased after 4.4 months (recurrent AML with DIC)

Tissue factor procoagulant activity (TF PCA), expressed as arbitrary units (AU), is of intact peripheral blood mononuclear cells. Reference ranges are as follows: platelets, 150–400 × 10^3^/µL; fibrinogen, 1.8–4.0 g/L; D-dimer, 0–0.5 mg/L; LDH, 84–246 U/L.

*ACS* acute coronary syndrome, *APL* acute promyelocytic leukemia, *Fg* fibrinogen, *ICH* intracerebral hemorrhage, *LDH* lactate dehydrogenase, *MP* microparticle, *PE* pulmonary embolism, *PB* peripheral blasts.

**Fig. 2:**
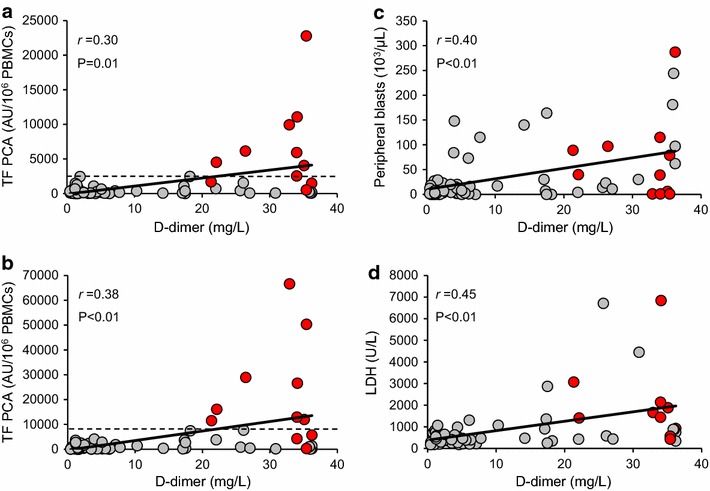
Correlations of PBMC-associated TF PCA, circulating blasts, and serum lactate dehydrogenase (*LDH*) with plasma D-dimer. Cellular TF PCA was measured by single-stage clotting assay using both intact (**a**) and disrupted PBMCs (**b**). Absolute numbers of circulating blasts (**c**) were calculated by multiplying the automatically determined white blood cell count with the percentage of myeloblasts on peripheral blood smears. LDH serum levels (**d**) were determined by standard laboratory techniques. Correlation coefficients and *P* values are according to the method of Spearman. The *horizontal dashed lines* in *panels* **a** and **b** indicate thresholds above which TF PCA levels were inevitably associated with decompensated DIC and a (thrombo)hemorrhagic syndrome as observed in eleven patients (*red data points*). The LDH serum level was not available from one patient with DIC.

In the total AML patient cohort (n = 69), plasma levels of D-dimer significantly correlated with TF PCA of intact (Fig. 2a) and disrupted PBMCs (Fig. 2b), absolute numbers of circulating blasts (Fig. 2c), and lactate dehydrogenase (LDH) serum levels (Fig. 2d). However, while expression of PBMC-associated TF PCA beyond a certain level (i.e. >2,500 AU/10^6^ intact PBMCs or >8,000 AU/10^6^ disrupted PBMCs) was inevitably associated with DIC, no such association was observed for peripheral blasts or LDH (Fig. 2).

**Fig. 3:**
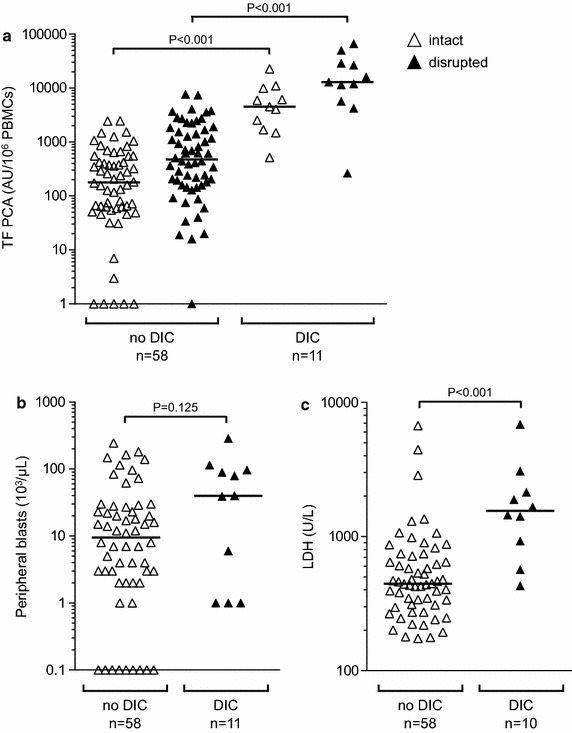
Evolution of DIC in newly diagnosed AML is associated with increased TF PCA and serum LDH. **a** TF PCA was measured by single-stage clotting assay using both intact and disrupted PBMCs. Peripheral blasts (**b**) and serum LDH (**c**) were determined as described above. For illustration purposes only, data points with “zero” peripheral blasts were plotted as 0.1/µL. *P* values are according to Mann–Whitney U test. The LDH serum level was not available from one patient with DIC.

Levels of PBMC-associated TF PCA were significantly increased in DIC patients (n = 11) as compared to non-DIC patients (n = 58), both before and after cell disruption (Fig. 3a). While there was no difference with regard to absolute numbers of circulating blasts between the two groups (Fig. 3b), patients with DIC had significantly higher LDH serum levels (Fig. 3c). These findings may indicate that, in addition to TF PCA expression by transformed myeloblasts, leukemic cell apoptosis/necrosis significantly contributed to the development of DIC in patients with newly diagnosed AML.

### TF PCA of plasma microparticles is associated with DIC

**Fig. 4:**
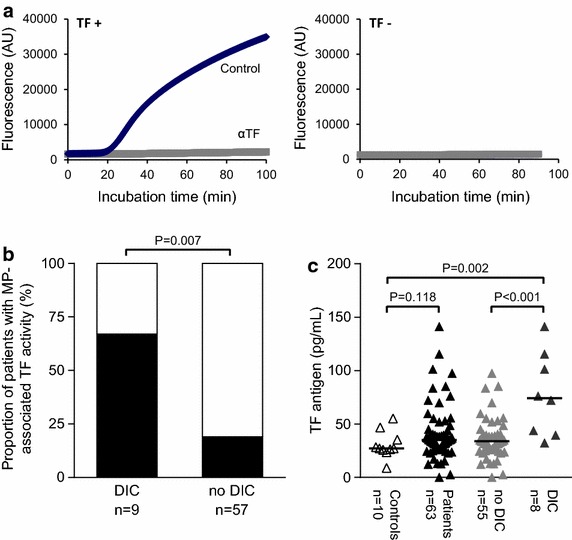
Microparticle-associated TF PCA and soluble plasma TF antigen are associated with decompensated DIC in newly diagnosed AML. Microparticles (*MPs*) were isolated from plasma and analyzed for TF PCA by thrombin generation assay as described under the section “Methods”. **a** Representative thrombin generation curves from two patients tested positive (*TF+*) or negative (*TF−*) for MP-associated TF PCA are shown (αTF, inhibitory TF antibody). **b** Proportions of patients tested positive for MP-associated TF PCA in the DIC and non-DIC cohorts are indicated by the *black columns*. *P* value is according to Fisher’s exact test. Plasma for MP isolation was not available from two patients with DIC and one patient without DIC. **c** A commercial ELISA was used to measure soluble TF antigen levels in plasma that was not available from three DIC and two non-DIC patients. *P* values are according to Mann–Whitney U test.

Because microparticles (MPs) are known to be released during apoptosis/necrosis, we measured circulating TF-bearing MPs in DIC and non-DIC patients using a qualitative thrombin generation assay (TGA) (Fig. 4a). Plasma for MP isolation was not available from two patients with DIC and one patient without DIC. TF-bearing plasma MPs were detectable in six out of nine DIC patients (67%) and in eleven out of 57 non-DIC patients (19%) (Fig. 4b). Interestingly, all three of the APL patients with DIC from whom plasma samples were available were tested positive for TF-bearing plasma MPs (Table 2), but not the one APL patient without DIC. These findings indicate that DIC evolution in newly diagnosed AML is associated with increased shedding of TF-bearing plasma MPs. We also measured soluble TF antigen in plasma samples available from 63 AML patients, including eight patients with DIC. While there was no significant difference in plasma TF antigen levels between AML patients (n = 63) and healthy controls (n = 10), DIC patients (n = 8) had significantly higher levels than non-DIC patients (n = 55) and controls (Fig. 4c), further indicating that TF expression is upregulated in AML-associated DIC.

### Cellular TF PCA is not correlated with VEGF secretion

**Fig. 5:**
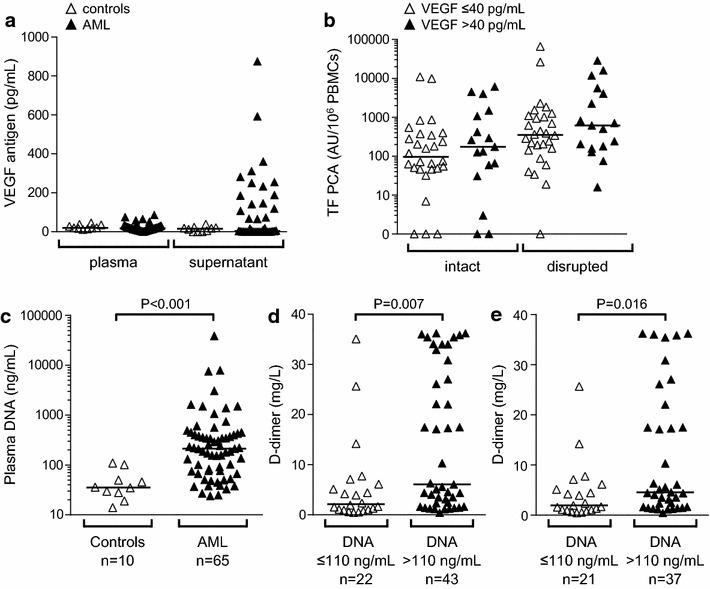
Associations of VEGF and cell-free plasma DNA with TF PCA expression and systemic coagulation activation. **a** Vascular endothelial growth factor (*VEGF*) antigen levels were measured in plasma or PBMC culture supernatants by ELISA. **b** Based on VEGF antigen levels in culture supernatants, AML patients were grouped into those with (>40 pg/mL) and those without (≤40 pg/mL) appreciable VEGF production. No significant differences were found with regard to TF PCA levels in intact or disrupted PBMCs. **c** Levels of cell-free plasma DNA were quantified in patients and controls as described under the section “Methods”. Plasma for DNA quantification was not available from four patients with AML. **d** Based on levels of cell-free plasma DNA, patients were grouped into those with DNA levels greater or equal or less than the cut-off value of 110 ng/mL defined by the 95th percentile within the control group. **e** In a modified analysis, the seven AML patients with DIC and TF PCA levels of intact PBMCs exceeding 2,500 AU/10^6^ cells were excluded. *P* values are according to Mann–Whitney U test.

Experimental and clinical evidence indicates that angiogenesis and coagulation activation (i.e. TF expression) are closely interrelated in patients with solid malignancies [12]. Because increased VEGF and bone marrow microvessel density have also been reported in AML [13–16], we explored such a relationship in our study cohort. To this end, VEGF antigen levels were measured in plasmas and culture supernatants, which were available from 46 and 45 of the AML patients, respectively. There was no difference in VEGF plasma levels between patients and controls (Fig. 5a). There was also no statistically significant difference in VEGF antigen levels in culture supernatants between the two groups. However, 17 of the studied 45 AML patients (38%) had VEGF antigen levels of >40 pg/mL, whereas all control subjects had VEGF antigen levels of <40 pg/mL (Fig. 5a). We used this arbitrary cut-off value to investigate, if VEGF secretion by isolated PBMCs was associated with cell-associated TF PCA, but no such association could be found, neither for intact nor for disrupted PBMCs (Fig. 5b).

### Potential role of cell-free DNA in intravascular coagulation activation

Recently, neutrophil extracellular traps (NETs) have been identified as activators of blood coagulation [17, 18]. Furthermore, NETs are associated with various cancer-associated clotting disorders [19], including deep-vein thrombosis and thrombotic microangiopathies [20–22]. DNA is a major component of NETs and sufficient to accelerate blood clotting [23]. We therefore asked if increased levels of cell-free DNA could account for systemic coagulation activation, as assessed by plasma D-dimer, in our AML patient cohort. Plasma for DNA quantification was not available from four AML patients, including three patients with DIC. Compared to healthy controls (n = 10), AML patients (n = 65) had significantly increased levels of cell-free plasma DNA (Fig. 5c). In AML patients, extracellular DNA levels significantly correlated with plasma D-dimer (*r* = 0.31, *P* = 0.011) and serum LDH (*r* = 0.37, *P* = 0.002), but not with peripheral blasts (*r* = 0.22, *P* = 0.080). To further explore the relevance of cell-free DNA for systemic coagulation activation, AML patients (n = 65) were grouped into those with plasma DNA levels of >110 ng/mL (n = 43), corresponding to the 95th percentile of plasma DNA levels within the control cohort, and those with plasma DNA levels of ≤110 ng/mL (n = 22). Patients with cell-free DNA levels above this cut-off value had significantly increased plasma D-dimer (Fig. 5d). In a subsequent analysis, we excluded all DIC patients with TF PCA levels of intact PBMCs exceeding 2,500 AU/10^6^ cells (n = 7), because in these patients, expression of TF PCA was considered the predominant cause of systemic coagulation activation (Fig. 2a). In this modified study population (n = 58), plasma DNA levels of >110 ng/mL were still associated with significantly increased D-dimer (Fig. 5e).

### Effect of TF PCA on clinical outcome

**Fig. 6:**
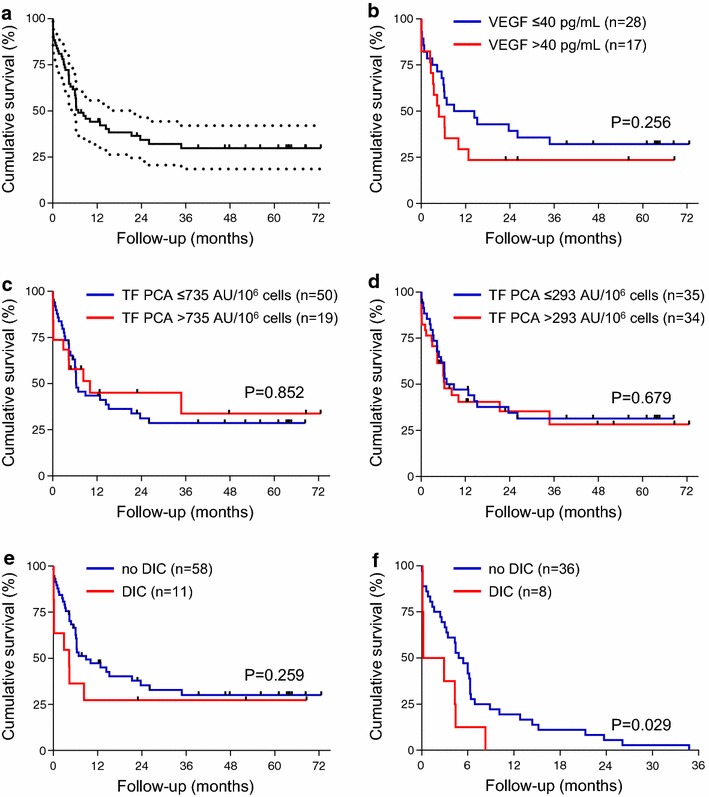
Impact of VEGF production, TF PCA expression and DIC evolution on overall survival. **a** Overall survival of the total AML patient cohort (n = 69). The dashed lines indicate the 95% confidence interval. **b** Overall survival of AML patients grouped according to VEGF antigen levels in culture supernatants. **c**, **d** Overall survival of AML patients grouped according to TF PCA levels of intact PBMCs. Cut-off values for TF PCA were defined as the 95th percentile within in the control group (**c**) or as the median within the patient cohort (**d**). **e** Overall survival of AML patients grouped according to the presence of DIC at presentation. **f** Survival analysis of DIC and non-DIC patients who died during the observation period.

In the total AML patient cohort (n = 69), median (mOS) and 5-year overall survival (OS) was 6.9 months and 29.9%, respectively (Fig. 6a). Increased VEGF expression has been associated with a more aggressive phenotype and an unfavorable clinical outcome in AML [14]. We therefore first analyzed our patient cohort with respect to ex vivo VEGF production. Patients with increased VEGF production (i.e. VEGF antigen in culture supernatant, >40 pg/mL) had a shorter mOS (4.8 vs. 11.7 months) and a decreased OS (23.5 vs. 32.1%) than patients with no appreciable VEGF production (i.e. VEGF antigen, ≤40 pg/mL) (Fig. 6b). Although this difference did not reach statistical significance, which was likely due to the relatively small sample size, it is consistent with the postulated role of VEGF in AML biology.

In contrast, no difference was found for mOS or OS, when AML patients were stratified according to the TF PCA level of intact PBMCs using either 735 AU/10^6^ cells (corresponding to the 95th percentile of TF PCA levels within the control group) or 293 AU/10^6^ cells (corresponding to the median TF PCA level within the patient cohort) as the cut-off value (Fig. 6c, d). While the presence of DIC at presentation did not adversely affect OS (Fig. 6e), it was significantly associated with early mortality (Fig. 6f).

## Discussion

In this study, we investigated PBMC-associated TF PCA in 69 patients with newly diagnosed AML and found that TF PCA expression beyond a critical level was inevitably associated with the evolution of decompensated DIC. Importantly, DIC was not restricted to APL patients, but also occurred in seven other patients, three of whom had acute (myelo)monocytic leukemia. To our knowledge, this is one of the largest prospective studies so far specifically addressing the determinants of systemic coagulation activation in newly diagnosed AML.

The increase in PBMC-associated TF PCA following cell disruption is consistent with the concept of cryptic TF that can be de-encrypted upon cellular activation or induction of apoptosis/necrosis [24]. Previous studies have clearly shown that cryptic TF is entirely cell surface-expressed on (myelo)monocytic cells and not liberated from intracellular storage pools upon cell lysis [25–27]. Instead, negatively charged phospholipids (i.e. phosphatidylserine) exposed on the outer membrane leaflet act synergistically with thiol-disulfide exchange reactions to activate surface-expressed TF [28–30]. In this regard, our findings provide circumstantial clinical evidence that membrane alterations associated with apoptosis/necrosis indeed contribute to cellular TF activation in vivo, because serum LDH levels, a surrogate marker for leukemic cell turnover, significantly correlated with plasma D-dimer. Systemic coagulation activation in AML is thus, at least to a substantial extent, determined by the following three factors: specific upregulation of cellular TF PCA following leukemic transformation of hematopoietic stem cells, elevated numbers of circulating blasts, and a significant degree of spontaneous apoptosis/necrosis. In our study, none of the patients developed overt DIC after the implementation of induction chemotherapy, which may de-encrypt cellular TF through the above mechanisms [26, 29]. Interestingly, in DIC patient no. 11 (Table 2), the systemic coagulopathy resolved during phases of AML remission, but recurred simultaneously with fulminant AML relapse, suggesting that the procoagulant phenotype of myeloblasts may be preserved despite intensive anti-leukemic therapy, including allogenic hematopoietic stem cell transplantation.

Our findings also point to a role of circulating TF-bearing MPs in the pathogenesis of AML-associated DIC, which has most recently been suggested by other experimental and clinical observations [31–33]. Shedding of MPs typically results from cellular activation or apoptosis, but may occur spontaneously in solid cancers and hematological malignancies [34]. Although MP-associated TF PCA was more frequently detected in DIC as compared to non-DIC patients, our findings do not allow the conclusion that shedding of TF-bearing MPs alone was sufficient to cause overt DIC in newly diagnosed AML. However, caution is warranted when comparing results of different studies due to a lack of standardized assays to measure MP TF.

The finding that plasma TF antigen was significantly increased in DIC as compared to non-DIC patients and healthy controls may be interpreted in favor of our conclusion that TF is an important determinant of DIC evolution in newly diagnosed AML. It is important to note, however, that solid-phase immunoassays may preferentially capture soluble (i.e. truncated) TF, which is functionally inactive, rather than MP-associated TF. Furthermore, there is concern about a lack of specificity when using human plasma in commercial TF ELISAs, which may result in inappropriately high background signals [35, 36]. Nevertheless, in our study, plasma TF antigen was specifically upregulated in patients with DIC (Fig. 4c). Because the commercial TF ELISA used in our study does not detect alternatively spliced TF (unpublished data), future studies should therefore investigate, if proteolytic degradation of full-length TF by enzymes liberated from dying myeloblasts represents a novel mechanism of controlling the extrinsic coagulation pathway in newly diagnosed AML. Taken together, functional assays are more sensitive and specific than ELISAs in detecting minute amounts of biologically active TF, but TF immunoassays may provide useful complementary information in complex disease states.

An important finding of our study is that significant systemic coagulation activation, as indicated by excessively elevated plasma D-dimer, may occur in the absence of TF PCA overexpression by PBMCs. In fact, three of the eleven DIC patients (27%) had TF PCA levels below the threshold values of 2,500 AU/10^6^ cells for intact and 8,000 AU/10^6^ cells for disrupted PBMCs (Fig. 2). All of these patients had quite high numbers of circulating blasts, but two of them had only moderately increased serum LDH levels (Table 2). These observations point to alternative cellular and molecular pathways contributing to systemic coagulation in newly diagnosed AML. In this regard, negatively charged polymers such as polyphosphates or nucleic acids (i.e. RNA and DNA) have recently been identified as key mediators of intrinsic contact pathway activation in vivo [37]. Consistent with a highly aggressive hematological malignancy leading to substantial spontaneous cell death, we found significantly increased levels of cell-free plasma DNA in the AML patient cohort. Furthermore, DNA levels correlated with plasma D-dimer and serum LDH. Even when the seven DIC patients with exceedingly high PBMC-associated TF PCA were excluded, levels of cell-free plasma DNA were associated with the degree of systemic coagulation activation (Fig. 5e). These findings may be regarded as circumstantial clinical evidence that liberation of DNA from apoptotic/necrotic AML blasts indeed contributes to intravascular thrombin generation through contact activation of factor XII [38] and may thus stimulate further research into the role of the intrinsic coagulation pathway in AML-associated coagulopathies. It has to be noted, however, that we did not specifically consider the contribution of cytoplasmic RNA [23], which may be liberated from dying cells at an earlier stage than nuclear DNA and which may be more closely correlated with serum LDH levels. Nevertheless, DNA-mediated activation of factor XII could explain why other studies did not find a significant contribution of the TF-dependent coagulation pathway to systemic coagulation activation in various hematological malignancies, including AML [9, 10]. Another potential mediator of PCA in AML is cancer procoagulant (CP), a cysteine proteinase that activates factor X independently of factor VIIa (FVIIa) [39], although more recent studies have questioned a predominant role of CP in paraneoplastic coagulation activation [40].

In solid malignancies, TF expression has been linked to tumor angiogenesis. In particular, TF has been shown to regulate VEGF production via its cytoplasmic domain [41], and TF-FVIIa-mediated cell signaling through cleavage of protease-activated receptor-2 (PAR-2) is crucial to neoplastic blood vessel formation [42]. Although such a relationship has also been postulated for hematological malignancies [43–45], our findings do not support an obvious association between TF PCA expression and VEGF secretion by isolated PBMCs in newly diagnosed AML. It has to be noted, however, that TF PCA, as measured in our study, is not directly coupled to TF signaling functions, because cryptic (i.e. non-coagulant) TF still binds FVIIa and thus retains its ability to activate PAR-2 [46]. Furthermore, measurement of ex vivo VEGF production by isolated PBMCs may not adequately reflect bone marrow microvessel density, which is also likely dependent on additional angiogenic growth factors (e.g. bFGF, HGF) not addressed in our study [16].

Patients presenting with DIC had a higher probability of early death (Fig. 6f). In this regard, our observation that TF PCA of both intact and disrupted PBMCs was associated with the presence of DIC may point to a prognostic role of TF in newly diagnosed AML. However, using either the upper limit of normal or the median TF PCA level of intact PBMCs as an arbitrary cut-off value, we did not observe an effect of increased TF PCA expression on median or OS in our patient cohort.

Our study has several limitations. First, although the total patient cohort is one of the largest so far, the generalizability of conclusions is still limited by the relatively small sample size of DIC patients. Likewise, much larger studies may be required to define the true impact of leukemic cell TF PCA expression on clinical outcome in newly diagnosed AML. Second, we used a qualitative TGA to measure MP-associated TF PCA. Although this assay may adequately mirror TF-driven coagulation activation in a plasma environment, quantitative assays such as a previously described chromogenic FXa generation assay [36] may provide more subtle information on the role of TF-bearing MPs in AML-associated DIC. Third, the impact of induction chemotherapy or treatment with all-trans retinoic acid on TF PCA expression and MP shedding was not addressed in our study. Finally, albeit novel, our findings on the association of cell-free DNA with plasma D-Dimer are rather descriptive, and mechanistic studies with functional readouts and different methods of DNA isolation and quantification are thus required to further define the role of intrinsic contact pathway activation in newly diagnosed AML.

## Conclusion

Our study clearly shows that PBMC-associated TF PCA expression beyond a threshold level inevitably results in decompensated DIC in patients with newly diagnosed AML. While shedding of TF-bearing MPs is likely involved in the pathophysiology of AML-associated coagulopathy, additional (i.e. TF-independent) pathways contribute to systemic coagulation activation and DIC evolution. Of these pathways, liberation of cell-free DNA may be a novel mediator of intrinsic contact pathway activation in this hematological malignancy.

## Methods

### Patients

The study protocol was approved by the ethics committee of the city of Hamburg, Germany (no. OB-059/05), and the study was conducted in accordance with the second Declaration of Helsinki. All patients and controls provided written informed consent.

Patients were eligible if they had been admitted to the University Medical Center Hamburg-Eppendorf, Germany, for diagnostic work-up and subsequent treatment of presumed AML, which was confirmed by standard cytological, cytogenetic, and immunophenotypic criteria in 69 patients (Table 1). In five and seven patients, however, the final diagnoses were ALL or MDS, respectively. These patients served as additional reference groups for the analysis of PBMC-associated TF PCA (see below). The ALL patient cohort comprised four females and one male (mean age, 37 ± 14 years), and the MDS patient cohort comprised three females and four males (65 ± 9 years). The group of healthy controls (n = 10) included four females and six males (54 ± 18 years). Although consecutive patient enrollment was not feasible for logistic or consenting reasons, patients were enrolled on a random basis between August 2005 and November 2014, thus grossly representing the population of AML patients treated at our institution.

Patients had to be at least 18 years of age with no clinical and laboratory evidence for acute bacterial or viral infections or severe hepatic or renal dysfunction. Major surgical procedures within the preceding 4 weeks and any systemic anticoagulation prior to study enrollment were exclusion criteria.

### Blood sampling and processing

All blood samples were taken before the initiation of specific anti-leukemic therapy. Venous blood was drawn by puncture of an antecubital vein or through a central venous catheter into plastic tubes prefilled with either 3.2% (0.109 M) sodium citrate or lithium heparin. Citrate-anticoagulated whole blood was centrifuged for 18 min at 4,000 rpm (1,200×*g*) to obtain platelet-poor plasma, which was snap-frozen in liquid nitrogen and stored in aliquots at −80°C until analysis. Heparin-anticoagulated whole blood was used to isolate peripheral blood mononuclear cells (PBMCs) as described below.

### Measurement of plasma D-dimer and thrombin–antithrombin (TAT) complexes

Plasma D-dimer and TAT complexes were quantified using the Innovance™ D-dimer test on a BCS™ coagulation analyzer and the Enzygnost™ TAT micro kit, respectively (Siemens Healthcare).

### Isolation of PBMCs

PBMCs were isolated from heparinized whole blood by density gradient centrifugation on Ficoll-Hypaque (Pharmacia) as previously described [26]. Cells were washed in phosphate-buffered saline (PBS), adjusted to 1 × 10^6^/mL, and immediately analyzed for TF procoagulant activity (PCA).

### Measurement of cellular TF PCA

A previously described single-stage clotting assay was used to measure TF-specific PCA in PBMCs that were either left intact or disrupted by repeated freeze-thawing [47]. Briefly, 100 µL of cell suspension (1 × 10^6^/mL) were incubated with 100 µL of normal human plasma (NHP) (HemosIL™; Instrumentation Laboratory) in the cuvettes of a KC10 coagulation instrument (Amelung) for 2 min at 37°C. Following the addition of CaCl_2_ (final concentration, 5 mM), times until fibrin clot formation were recorded. All measurements were carried out in triplicates in the presence of 30 µg/mL inhibitory TF monoclonal antibody (#4509; American Diagnostica) or control IgG (Sigma). Clotting times were converted into arbitrary PCA units (AU) by reference to a standard curve obtained by serial dilutions of lipidated recombinant human TF (Innovin™; Dade Behring).

### Measurement of microparticle-associated TF PCA

Plasma microparticles (MPs) were isolated by centrifugation at 100,000×*g* for 90 min at 10°C. Pellets were resuspended in the original volume with PBS. MP-associated TF activity was measured by a TGA according to the method developed by Hemker [48, 49]. Briefly, 20 µL of MP suspension were added, along with 20 µL of a mixture containing CaCl_2_ and the fluorogenic substrate Z-GGA-AMC-HCl (Bachem BioSciences), to 80 µL NHP in the presence of 25 µg/mL corn trypsin inhibitor (Enzyme Research Laboratories). Thrombin generation was measured over a period of 90 min using a Synergy 2 fluorometer (BioTek). MP suspensions were added as the only source of TF and phospholipids to initiate coagulation. Inhibitory TF antibody H36 (50 µg/mL) (Sunol Molecular) was employed to verify TF specificity.

### Measurement of plasma TF antigen

Plasma TF antigen was measured using the Quantikine™ enzyme-linked immunosorbent assay (ELISA) (R&D Systems) according to the manufacturer’s instructions.

### Measurement of VEGF antigen in plasma and culture supernatant

The Quantikine™ ELISA (R&D Systems) was used to measure vascular endothelial growth factor (VEGF) antigen levels in plasma samples and culture supernatants. Supernatants were prepared by incubating isolated PBMCs (1 × 10^6^/mL) for 24 h at 37°C and 5% CO_2_ in RPMI culture medium in the presence of 10% fetal calf serum. Cells were removed by centrifugation and supernatants stored at −80°C until analysis.

### Quantification of cell-free plasma DNA

Plasma DNA was quantified based on a previously described method [22]. Briefly, 2 µL of plasma were diluted in 98 µL of PBS containing 0.1% bovine serum albumin. Diluted plasma was then mixed with 100 µL PBS containing Sytox™ Green nucleic acid stain (Invitrogen) at a final concentration of 2 µM to label DNA fluorescently. Fluorescence was recorded using MTP reader (Tecan) with a 485 nm excitation and 535 nm emission filter set. Autofluorescence was considered as background and determined in samples mixed with PBS without Sytox™ Green. DNA concentrations were calculated based on a standard curve obtained by known concentrations of DNA.

### Statistical analysis

Normally and non-normally distributed data were presented as mean ± standard deviation and median with (interquartile) range, respectively. Differences between two groups were analyzed using the two-sided Student’s *t* test or the Mann–Whitney U test. Differences between multiple groups were analyzed using the Kruskal–Wallis test. Categorical data were analyzed using the Fisher’s exact test. Correlation coefficients were according the method of Spearman. Kaplan–Meier survival curves were compared using the log-rank (Mantel–Cox) test. All analyses were carried out using GraphPad Prism™ software. A *P* value of <0.05 was considered statistically significant.

## Abbreviations

ACS: acute coronary syndrome
ALL: acute lymphoblastic leukemia
AML: acute myeloid leukemia
APL: acute promyelocytic leukemia
AU: arbitrary units
bFGF: basic fibroblast growth factor
CP: cancer procoagulant
DIC: disseminated intravascular coagulation
DNA: deoxyribonucleic acid
ELISA: enzyme-linked immunosorbent assay
FAB: French–American–British
Fg: fibrinogen
HGF: hepatocyte growth factor
ICH: intracerebral hemorrhage
ISTH: International Society on Thrombosis and Haemostasis
LDH: lactate dehydrogenase
MDS: myelodysplastic syndrome
mOS: median overall survival
MP: microparticle
MPN: myeloproliferative neoplasia
NETs: neutrophil extracellular traps
NHP: normal human plasma
OS: overall survival
PAR: protease-activated receptor
PB: peripheral blasts
PBMCs: peripheral blood mononuclear cells
PBS: phosphate-buffered saline
PCA: procoagulant activity
PE: pulmonary embolism
PS: phosphatidylserine
RNA: ribonucleic acid
SSC: Scientific and Standardization Committee
TAT: thrombin–antithrombin
TF: tissue factor
TGA: thrombin generation assay
VEGF: vascular endothelial growth factor
